# S100A7-Downregulation Inhibits Epidermal Growth Factor-Induced Signaling in Breast Cancer Cells and Blocks Osteoclast Formation

**DOI:** 10.1371/journal.pone.0001741

**Published:** 2008-03-05

**Authors:** Vikram Paruchuri, Anil Prasad, Kevin McHugh, Hari K. Bhat, Kornelia Polyak, Ramesh K. Ganju

**Affiliations:** 1 Division of Experimental Medicine, Beth Israel Deaconess Medical Center, Harvard Medical School, Boston, Massachusetts, United States of America; 2 Division of Rheumatology, Beth Israel Deaconess Medical Center, Harvard Medical School, Boston, Massachusetts, United States of America; 3 Department of Environmental Health Sciences, Mailman School of Public Health, Columbia University, New York, New York, United States of America; 4 Dana-Farber Cancer Institute, Harvard Medical School, Boston, Massachusetts, United States of America; Ordway Research Institute, United States of America

## Abstract

S100A7 is a small calcium binding protein, which has been shown to be differentially expressed in psoriatic skin lesions, as well as in squamous cell tumors of the skin, lung and breast. Although its expression has been correlated to HER+ high-grade tumors and to a high risk of progression, the molecular mechanisms of these S100A7-mediated tumorigenic effects are not well known. Here, we showed for the first time that epidermal growth factor (EGF) induces S100A7 expression in both MCF-7 and MDA-MB-468 cell lines. We also observed a decrease in EGF-directed migration in shRNA-downregulated MDA-MB-468 cell lines. Furthermore, our signaling studies revealed that EGF induced simultaneous EGF receptor phosphorylation at Tyr1173 and HER2 phosphorylation at Tyr1248 in S100A7-downregulated cell lines as compared to the vector-transfected controls. In addition, reduced phosphorylation of Src at tyrosine 416 and p-SHP2 at tyrosine 542 was observed in these downregulated cell lines. Further studies revealed that S100A7-downregulated cells had reduced angiogenesis *in vivo* based on matrigel plug assays. Our results also showed decreased tumor-induced osteoclastic resorption in an intra-tibial bone injection model involving SCID mice. S100A7-downregulated cells had decreased osteoclast number and size as compared to the vector controls, and this decrease was associated with variations in IL-8 expression in *in vitro* cell cultures. This is a novel report on the role of S100A7 in EGF-induced signaling in breast cancer cells and in osteoclast formation.

## Introduction

S100A7 (also known as psoriasin) is a small calcium binding protein of 11 kDa molecular weight, first described as an mRNA expressed in psoriatic skin lesions [1]. It is a member of the S100 family of the EF-hand type of calcium binding proteins. The S100A7 protein is known to be expressed in various tumors having squamous differentiation as a major component with or without accompanying inflammation (eg, squamous cell carcinoma of the skin [2], [3], lung [4], cervix, bladder [5] and breast as well as adenocarcinoma of the breast [6]. S100A7 was identified as a differentially expressed gene in ductal carcinoma in-situ (DCIS) but not in invasive breast carcinomas, suggesting its potential role in tumor progression. Expression of S100A7 has been shown to be correlated with HER+, high-grade tumors [6]. The high expression level of S100A7 in poorly differentiated and lymph node positive breast tumors suggests that it may predict poor clinical outcome and a high risk of recurrence or progression in DCIS [7]. Although S100A7 has been reported to play a role in breast cancer, the molecular mechanisms of its effects are not well known. Recent studies have suggested that EGF may regulate S100A7 expression [8].

EGF and its related family member, HER2/Neu, are commonly expressed in breast cancers, including in 60% of invasive breast cancers. Overexpression of HER2 was previously linked to DCIS [9]. In addition, overexpression of EGF was correlated with tumor progression and extensive metastasis in breast cancers [10], and other malignancies [11]. Breast carcinomas with squamous differentiation are a distinct subgroup of breast tumors with a high frequency of EGF receptor positivity [12]. EGFR is a 170 kDa Type 1 transmembrane glycoprotein containing an extracellular ligand-binding domain, transmembrane domain, and a cytoplasmic tail, which includes a tyrosine kinase domain and docking sites for binding [13].

Tumor angiogenesis plays an important role in tumor growth and metastasis. In the past two decades, numerous positive and negative regulators of angiogenesis have been described, the most recent one being VEGF. High VEGF levels have been detected in S100A7-overexpressing cells and these levels were correlated with increased tumor angiogenesis in human breast tumors [14].

The bone is the first site of metastasis in 25–50% of breast cancer cases and osteolytic lesions are present in 70–80% of patients with stage IV breast cancer [15], [16]. Histological analysis and scanning microscopy have revealed that bone destruction is mediated by osteoclasts. Tyrosine kinase inhibitors of EGFR have been shown to efficiently block the *in vitro* and *in vivo* activation of this receptor, and to significantly inhibit tumor growth in experimental animal models. Tumor cells, osteoclasts, stromal cells and the extracellular matrix are components required for the initiation and development of bone metastasis. Tumor cells activate osteoclasts via PTHrP, IL-6, IL-1, and TNF-α. PTHrP-independent factors like IL-11 and IL-8 also contribute to osteolytic activity [17]. Moreover, IL-8 is a major osteolytic factor and potent activator of bone destruction accompanying metastatic bone disease [18].

Our study for the first time reveals that S100A7 may regulate EGF-induced EGFR phosphorylation and other downstream signaling molecules. We found that S100A7-downregulated breast cancer cells exhibited a reduction in EGF-induced chemotaxis and invasion on matrigel-coated transwells. Furthermore, we showed that S100A7 reduced the number and size of osteoclasts formed *in vitro* and the size of osteolytic lesions observed *in vivo*. We also noted decreased angiogenesis in matrigel assays utilizing S100A7-downregulated cells. This is a novel report indicating the role of S100A7 in EGFR signaling and osteoclast resorption.

## Results

### Growth factors regulate S100A7 expression

**Figure 1:**
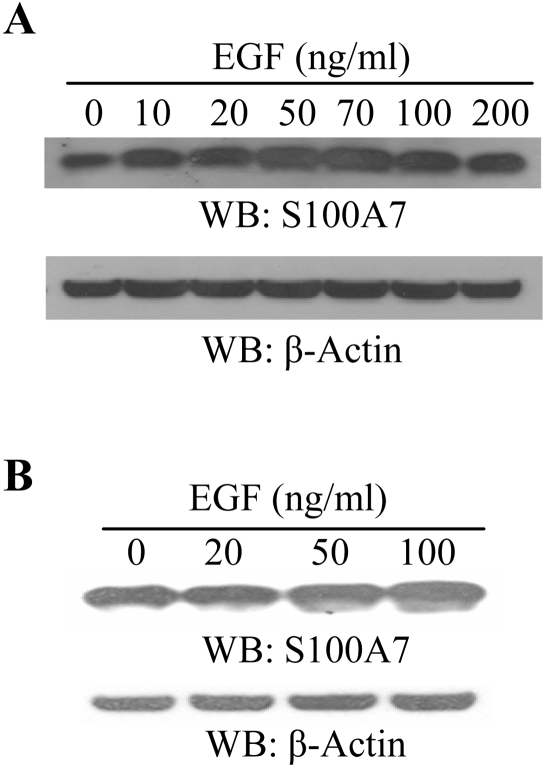
EGF stimulation enhances S100A7 expression in breast cancer cells. 70–80% confluent MCF-7 cells (A) and MDA-MB-468 cells (B) were washed twice and rinsed with PBS followed by 18 hrs of serum starvation. The cells were then stimulated with various concentrations of epidermal growth factor (EGF) for 30 minutes at 37°C. The cells were lysed and S100A7 expression was analyzed by Western blotting with anti-S100A7 antibody, as indicated. Equal protein loading in each lane was checked by stripping the blots and probing with β-Actin antibody (A and B, lower panels). The experiments were repeated thrice with identical results.

Recently, it has been shown that S100A7 is associated with an increase in EGF mRNA levels [8]. However, not much is known about growth factor effects on S100A7 expression in malignant breast tissues. Thus, we analyzed the effect of EGF on S100A7 expression in MCF-7 and MDA-MB-468 breast cancer cell lines. EGF increased the expression of S100A7 in a dose-dependent manner in both MCF-7 cells (Figure 1A) and MDA-MB-468 cells (Figure 1B). Peak effect was seen between 70–100 ng/ml of EGF.

### S100A7 silencing by S100A7 shRNA inhibits EGF-induced cell migration and invasion

**Figure 2:**
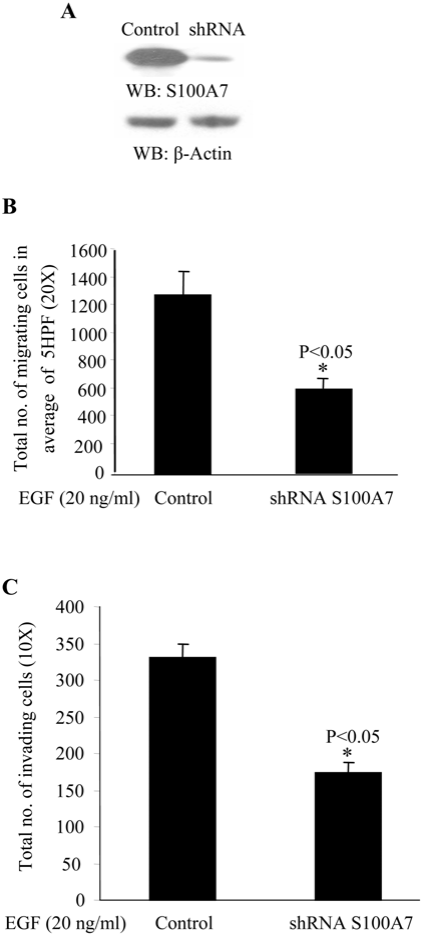
S100A7 knocked-down MDA-MB-468 cells show a decrease in EGF-induced migration and invasion. (A) MDA-MB-468 cells were transfected with vector control or S100A7-shRNA, as described in Materials and Methods. S100A7 expression in these cells was analyzed by Western blotting with anti-S100A7 antibody (upper panel). Equal protein loading in each lane was checked by stripping the blot and probing with β-Actin antibody (lower panel). (B) Control and S100A7 shRNA-transfected MDA-MB-468 cells were subjected to a chemotaxis assay towards EGF (20 ng/ml) using the modified Boyden chamber assay, as described in Materials and Methods. The lower surface of the insert was stained with the Diff-Quik Stain kit and cells were counted in an average of 5 high-power fields (HPF; 20×). Experiments were done in triplicate and repeated three times with similar results. *p<0.05 (C) Control and S100A7 shRNA-transfected MDA-MB-468 cells were subjected to an invasion assay using matrigel-coated transwell plates (BD Biosciences). Chemotaxis towards EGF (20 ng/ml) was then analyzed, as described in Materials and Methods. The cells on the insert were stained with the Diff-Quik Stain kit and total cell numbers were counted (10×). Experiments were done in triplicate and repeated thrice with similar results. *p<0.05.

To investigate the involvement of S100A7 in the EGF-mediated effects on migration and invasion, we used stable S100A7-shRNA-downregulated cells (Figure 2A), as previously reported [14]. We observed a reduction in both EGF-induced cell migration on fibronectin-coated plates in Boyden chambers (Figure 2B) and invasive activity on matrigel-coated plates (Figure 2C). The migration of the S100A7-downregulated cells towards EGF was significantly reduced (by 40%) as compared to the vector-transfected controls.

### S100A7 mediates EGFR/HER2 cell signaling through distinct and specific phosphorylation of tyrosine residues and downstream cascading events

**Figure 3:**
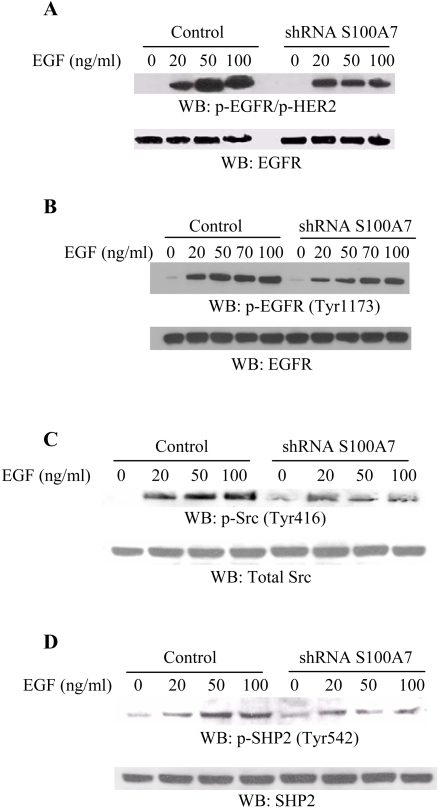
S100A7 downregulation decreases the phosphorylation of EGFR/HER2, Src and SHP2 in MDA-MB-468 cell lines. Vector control and S100A7-shRNA stably-transfected MDA-MB-468 cell lines were starved overnight and stimulated with various concentrations of EGF (0 to 100 ng/ml) for 30 minutes at 37°C. The cells were then lysed and the lysates were analyzed by Western blotting with p-EGFR Tyr1173/p-HER2 Tyr1248 (A, upper panel) or p-EGFR Tyr1173 (B, upper panel) antibodies. The protein levels were monitored by stripping the blots with anti-EGFR antibody (A and B, lower panels). Vector-transfected controls and S100A7 shRNA-transfected stable MDA-MB-468 cell lines were starved overnight for 17 hours and stimulated with various concentrations of EGF (0 to 100 ng/ml) for 15 minutes at 37°C. The cells were then lysed and the lysates were analyzed by Western blotting with p-Src Tyr416 (C, upper panel) or p-SHP2 Tyr542 antibody (D, upper panel). The protein levels were monitored by stripping the blots with total Src and SHP2 antibodies, as indicated (C and D, lower panels).

In order to evaluate the role of S100A7 in EGF-mediated signaling, we compared EGFR phosphorylation as well as the activities of various downstream signaling molecules in S100A7-vector control and S100A7-downregulated cell lines. Previously, it was shown that on ligand binding, EGFR activation involves homo- and hetero-dimerization with other EGFR family members (such as HER2), transphosphorylation of receptors, and activation of a number of different downstream signaling pathways [19]–[21]. EGFR expression has also been linked to activation of ErbB2 in human breast cancers [22]. In our study, phosphorylations of EGFR Tyr1173 and HER2 Tyr1248 (Figure 3A), EGFR Tyr1173 (Figure 3B), and Tyr1068 (data not shown) were reduced in S100A7-downregulated cells as compared to empty vector-transfected cell lines following treatment with increasing concentrations of EGF. A peak effect on phosphorylation was observed between 70–100 ng/ml of EGF. EGFR expression combined with c-Src overexpression can initiate oncogenic transformation and marked migratory and invasive behavior in human breast cancer cells [23]. We observed a decrease in Src phosphorylation at Tyr416 in EGF-stimulated S100A7-downregulated cells as compared to the control cells (Figure 3C). Moreover, since the tyrosine phosphatase SHP2 acts as an important positive regulator of EGFR signaling and is associated with signals initiated by receptor tyrosine kinases, we next evaluated SHP2 phosphorylation in the EGF-stimulated S100A7-downregulated cell lines as compared to the empty vector controls. SHP2 phosphorylation at Tyr542 was reduced in the S100A7-downregulated cells (Figure 3D). These results indicate that S100A7 alters EGF downstream signaling pathways.

### S100A7 downregulation reduces angiogenesis in a matrigel plug assay

**Figure 4:**
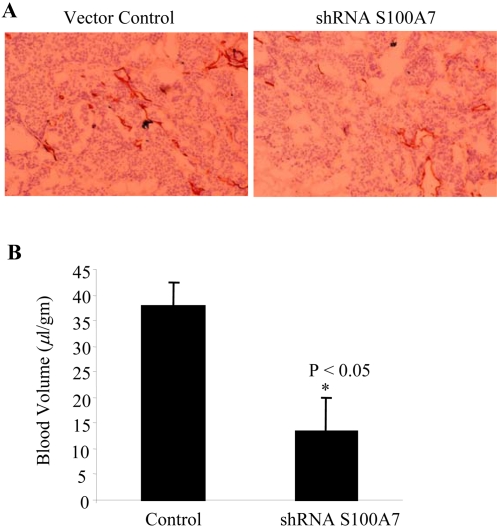
S100A7-downregulation decreases angiogenesis. (A) Effect of S100A7 on blood vessel formation in matrigel plugs containing MDA-MB-468 cell lines. 250 µl of Growth factor-reduced Matrigel Matrix High Concentration (BD Biosciences) was mixed with 6×10^6^ cells (250 µl of vector control or 250 µl of S100A7-downregulated cells) and implanted subcutaneously into SCID mice (two sites per mouse). After 4 weeks, one matrigel plug from each mouse injected with vector control or S100A7-shRNA cells was fixed in Tissue Tek and snap-frozen. The sections were stained for CD31 antibody by immunohistochemistry. (B) The other plug was grounded and the supernatant was analyzed for hemoglobin content using the Hemoglobin Assay Kit (Sigma Diagnostics). The results are interpreted as the blood volume per unit weight of matrigel (* p<0.05).

It has been shown that S100A7 overexpression increases VEGF expression levels (8) and that downregulation of S100A7 decreases the expression of VEGF [14]. In addition, a correlation was observed between high S100A7 expression and the angiogenic marker CD31 in samples derived from human breast cancer [14]. Thus, we examined the effect of S100A7 on angiogenic potential in SCID mice by using a matrigel plug assay. After 4 weeks, the matrigel plugs were either fixed for histology or extracted for hemoglobin. Evaluation of the matrigel plugs revealed a decrease in the number of blood vessels in the plugs from the S100A7-downregulated cells versus the control plugs, as detected by CD31 staining (Figure 4A). The inhibited blood vessel formation also resulted in a significant decrease in blood volume in the S100A7-downregulated cells containing the plugs, as measured by hemoglobin assays (Figure 4B).

### Downregulation of S100A7 decreases bone osteolysis

**Figure 5:**
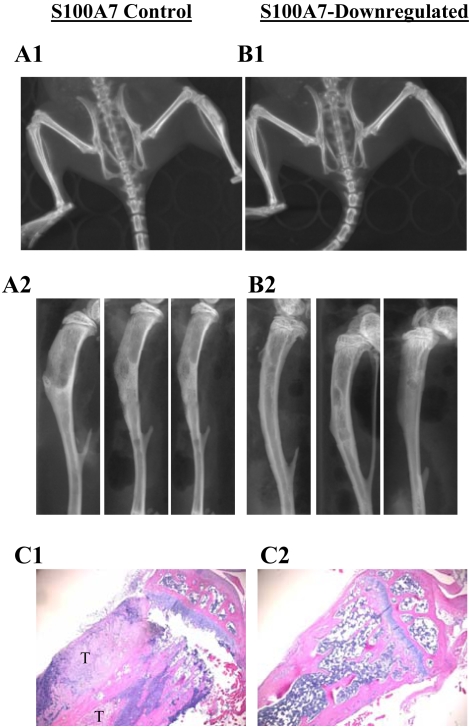
S100A7 downregulation decreases osteolytic lesions *in vivo.* Cells were directly introduced into the tibias of 4 week-old mice, as described in Materials and Methods. Micro-CT scanning of live mice was done using a GE eXplore Micro-CT scanner (GE Healthcare Ltd, UK) at 93 µm resolution, 6 weeks after injection. The tibias were dissected out and scout view radiographs were taken with a micro-CT40 scanner at 12 µm resolution obtained from SCANCO Medical AG (Switzerland). There were osteolytic lesions, destruction of the cortex and extension of the tumor into the soft tissue in the mice injected with the control S100A7 cells (Panels A1 and A2). The cortex of the mice injected with the S100A7-downregulated cells was intact (Panels B1 and B2). Tibias were excised and processed for conventional histological examination. S100A7 control mice showed tumor-induced osteolysis (Panel C1), and the osteolysis was evident in all three injected mice as compared to mice injected with the S100A7-downregulated cells (Panel C2) (H & E staining, 20×). The letter ‘T’ in panel C1 represents tumor. The experiments were repeated twice with identical results.

It has been shown that downregulation of S100A7 expression markedly decreases *in vivo* tumorigenicity and lung metastasis [14]. However, the involvement of S100A7 in the development of metastatic bone lesions and osteoclast formation at the site of metastasis is not known. Breast cancer cells that metastasize to the bone commonly cause osteolytic lesions, which are mediated by osteoclasts. Direct injection of cancer cells into mouse bone has been used as a model to analyze bone metastasis in renal carcinoma [24] and prostate cancer [25]. Here, we employed the orthotopic bone implantation model to analyze the formation of osteolytic lesions in mice. Intra-tibial injections in mice were carried out as described in Materials and Methods. We observed osteolytic lesions as well as destruction of the cortex, with tumors extending into the soft tissue in all three mice injected intra-tibially with the S100A7-control cells (Figure 5, Panels A1 and A2). The cortex on both sides was intact in mice injected with the S100A7-downregulated cells (Figure 5, Panels B1 and B2). H & E sectioning showed loss of the trabecular meshwork in the proximal tibia in the control group as compared to the S100A7-downregulated group (Figure 5: Panels C1 and C2, respectively). This result suggests that S100A7 may play a role in tumor-induced bone resorption.

### S100A7 downregulation decreases osteoclast formation in transwell assays

**Figure 6:**
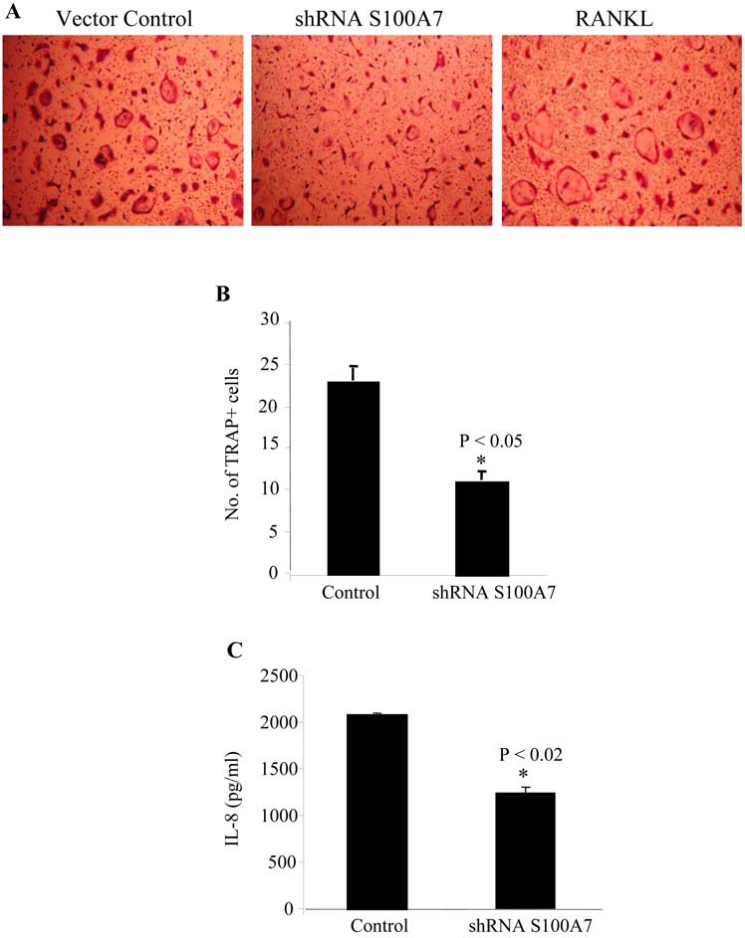
S100A7-downregulated cells decrease osteoclast formation and IL-8 expression levels. Osteoclast precursors were prepared (as described in Materials and Methods), plated in 24-well plates and allowed to stand for 5–6 hours. (A) Inserts containing 5×10^4^ vector control cells, the S100A7 shRNA (downregulated) cells or RANKL without breast cancer cells (internal positive control) were then placed on the wells and the numbers of TRAP+ multinucleated cells generated under each condition were counted at the end of 48 hours. Each culture was done in triplicate. The experiments were repeated thrice with similar results. (B) The number of multinucleated fused osteoclast cells (A) in an average of triplicate wells is represented in the histogram. Results are expressed as the mean±SEM (*p<0.05). (C) Vector control and S100A7 shRNA-transfected stable MDA-MB-468 cell lines were grown to 80% confluence and supernatants were analyzed for IL-8 expression by ELISA. IL-8 expression was significantly less (*p<0.02) in the S100A7-downregulated cells as compared to the vector-transfected controls.

Since we observed reduced osteolytic lesions in mice injected with S100A7-downregulated cells, we analyzed the role of S100A7 in osteoclast formation. Osteoclasts are known to mediate osteolytic lesions at the sites of metastatic tumors. Precursor osteoclasts, prepared from bone marrow cultures (as described in Materials and Methods), were placed in 24-well plates and inserts containing vector control or S100A7-shRNA-downregulated breast cancer cells (5×10^4^ cells) were placed in the wells. Addition of RANKL without breast cancer cells served as a control. We observed a marked increase in osteoclast formation and fusion as measured by the number and size of TRAP+ multinucleated (>3 nuclei) giant cells in the S100A7 control cells as compared to the downregulated cells (Figure 6A and B).

### Reduced IL-8 expression is present in S100A7-downregulated cell lines

Tumor cells secrete IL-8 which is known to stimulate the formation of osteoclasts from hematopoietic precursors in the bone marrow, resulting in increased osteoclastic bone resorption [18]. Furthermore, metastatic cells isolated from bone have been shown to produce greater amounts of IL-8 [17]. Therefore, we analyzed IL-8 production in the conditioned media of control and S100A7-downregulated cell lines. We observed a statistically significant decrease in IL-8 levels in the conditioned media of S100A7-downregulated cells as compared to S100A7 vector control cells (Figure 6C).

## Discussion

S100A7 is a member of the S100 family of proteins, which have been associated with preinvasive DCIS. Persistent expression of S100A7 occurs in some invasive cancers and is associated with poor prognostic factors [6]. Persistent S100A7 expression also occurs in a subset of invasive breast carcinomas and is linked to worse clinical outcome [26]. S100A7 has been shown to be overexpressed in breast cancers at sites of necrosis in tumor tissues [6], [8], as well as in the nasal fluid during allergic inflammatory reactions [27]. Although S100A7 has been reported to increase EGF mRNA levels in MDA-MB-231 cells [8], not much is known about its role in EGF-induced EGFR signaling. In the present study, we have shown that S100A7 modulates EGFR-induced phosphorylation, migration and functional effects. We also characterized EGFR's influence on S100A7 expression and its role in the EGF-induced metastasis and invasion of breast cancer cells.

Our experiments show the importance of S100A7 in EGF-induced and EGFR-mediated expression, signaling and migration. We have seen increased expression of S100A7 in two breast cancer cell lines following EGF stimulation. We observed a significant inhibition of EGF-induced migration on fibronectin-coated plates in S100A7-downregulated cell lines. There was also decreased EGF-directed invasion on matrigel-coated transwells. The results are in agreement with previous studies which reported a 1.4-fold increase in invasiveness in S100A7-overexpressing MDA-MB-231 clones [28]. However, different results were reported by other investigators [14] who observed an increase in the migration and invasion of S100A7-downregulated cells as compared to vector control cells. The absence of growth factors or serum in the above experiments could explain the differences in cell migration and invasion observed in those studies.

EGFR-mediated signaling has been correlated with disease stage and the development of tumor metastasis in breast cancer. We have observed that S100A7 regulates EGF-induced signaling. We noted a decrease in EGFR/HER2 phosphorylation at Tyr1068, Tyr1173, and Tyr1248 (HER2) sites in S100A7-downregulated MDA-MB-468 cells as compared to empty vector-transfected control cells upon EGF stimulation. Tyr1173 corresponds to the main site of EGFR autophosphorylation and has been shown to be critical for the tyrosine kinase activity of EGFR as well as its ability to mediate actions in response to EGF [29]. Tyr1068 and Tyr1248 are considered to be major *in vitro* autophosphorylation sites of EGFR and HER2, respectively [30]. Furthermore, Tyr1173 (EGFR) is known to be involved in radio-resistance and has also been shown to phosphorylate key intracellular molecules like GAB1, SHC and PLC-γ, which play a role in cell survival and migration in concert with HER2 receptors. Both HER2 and EGFR tyrosine kinase activation are known to predict the efficacy of trastuzumab-based therapy in patients with metastatic breast cancer [31].

The SHP2 tyrosine phosphatase acts as an important positive regulator of EGFR signaling [32]. We observed a dose-dependent decrease in SHP2 phosphorylation (at Tyr542) in EGF-stimulated S100A7-downregulated cell lines as compared to empty vector controls (Figure 3D). Tyr542 is the major Grb2 binding site in tyrosyl-phosphorylated SHP2. Phosphorylation of SHP2 at Tyr542 has been reported to stimulate its activity by 3-fold [33]. It has also been shown that SHP2 is necessary for ErbB2 to activate c-Src. Elevated Src expression is frequently observed in human breast cancer tissues compared with benign breast tumors or adjacent normal breast tissues and this elevated Src activity has been correlated with high grade, high proliferation and HER2 positivity in DCIS [34] as well as with poor metastasis-free survival in invasive breast cancers [35], [36]. Reduction in the amount of c-Src and subsequently that of EGFR activity has been shown to decrease invasion [37]. We have seen decreased phosphorylation of c-Src at Tyr416 in S100A7-downregulated cell lines as compared to vector-transfected controls. Tyr416 of Src is located in the kinase domain and phosphorylation of Src at this site enhances its catalytic activity. Thus, these data support a role for S100A7 as a modulator of the growth effects of EGF, which may have clinical implications in a subset of breast carcinomas where EGFR is overexpressed.

Suppression of EGFR signaling by EGFR inhibitors is known to reduce the incidence of prostate cancer metastasis to the bone in nude mice models [25]. The bone is a common site of metastases from breast, prostate, renal and thyroid cancers. Tumor growth in the bone depends on osteoclastic activity, which increases bone resorption and releases growth factors sequestrated in the bone matrix [38]. Most (∼85%) of breast cancer lesions are osteolytic in nature [39], in contrast to bone secondaries from prostate cancer which are osteosclerotic. Tumor cells induce these lesions directly by secreting factors that increase mature osteoclasts in the bone or indirectly through stromal cells, which secrete growth factors and cytokines causing the differentiation of precursors to active osteoclasts [16], [40]. Since the MDA-MB-468 cell line is not highly metastatic, it did not produce bone metastasis or gross visible lung metastasis on intravenous injections in our study. Therefore, we injected vector control cells and S100A7-downregulated cells into the tibia of mice to determine if S100A7 is involved in the osteoclast resorption of bone. We observed significant lytic lesions in the tibia with destruction of the cortex on CT imaging in control mice and few lytic lesions in mice injected with the S100A7-downregulated cells. The marrow and trabecular bones were destroyed in the proximal tibia of the control mice as compared to the tibia from mice with S100A7-downregulation, where the marrow cavity and trabecular bones remained intact. This suggests that S100A7 may play an important role in the formation of osteolytic lesions and hence in the metastasis of breast cancer cells to bone.

The development and progression of osteolytic lesions in the bone is a complex phenomenon involving tumor cell interaction with different cell types of the bone microenvironment [41]. We used an *in vitro* model involving osteoclast activation by breast cancer cells to confirm our *in vivo* findings. Human osteoclast precursors, isolated from mouse bone marrow, were differentiated into TRAP+ multinucleated cells, which were larger in size and number in the S100A7-control breast cancer cells as compared to the S100A7-downregulated cells in transwell assays. Breast cancer cells are able to synthesize many growth factors and cytokines that can lead to the activation of osteoclasts [38]. Several cytokines have been implicated in the invasiveness of breast tumors [42]. Low expression of IL-8 was seen in the supernatants of S100A7-downregulated cells. High IL-8 expression has been correlated with increased invasiveness and angiogenesis. Expression of IL-8 was also correlated with increased bone metastasis in a population of breast cancer cells [18], and tumors isolated from bone metastatic sites were reported to show high IL-8 secretion [43]. The latter was mediated through COX-2. In addition to the above *in vivo* findings, IL-8 has been demonstrated to have direct stimulatory effects on human osteoclastogenesis and bone resorption [17]. Breast cancer cells with high VEGF expression also have high IL-8 expression levels [44], which correlates with our data showing increased tumor growth and aggressiveness in S100A7-control cell lines as compared to S100A7-downregulated cell lines. Our *in vitro* results support our *in vivo* findings of increased osteolytic lesions in the S100A7 control group.

We observed that S100A7 expression was associated with increased blood vessel density in mouse models of human breast cancer. We demonstrated a reduction in angiogenesis *in vivo* as determined by decreased microvascular density in the histological sections of matrigel plugs containing S100A7-downregulated cells and high-concentration growth factor-reduced matrigel as compared to the S100A7-vector control cells when injected into mice. Our *in vivo* findings concur with a previous study which reported that S100A7-downregulated tumors have reduced VEGF expression *in vitro* as compared to vector-transfected control cells, and that human breast cancer tissues with decreased S100A7 expression levels have reduced CD31 staining [14]. Inhibition of blood vessel formation resulted in a significant decrease in blood volume in the S100A7-downregulated plugs. This was due to the reduced size of the tumor. However, when we compared blood volume per gram of tissue, the volume was decreased by 60% in the plugs from mice with S100A7-downregulation. Increased microvascular density has been correlated with active EGFR, tumor-type, tumor grade, and VEGF expression but not with HER2 expression in breast tumors [45]. ErbB2 has been shown to increase VEGF protein production in cancer cell lines, xenografts, and in human cancers [46].

S100A7 serves as an important growth regulatory protein in primary breast malignancies and could possibly control the cellular responses to EGF partly via the EGFR-dependent actions of EGF. We have found that epidermal growth factor regulates S100A7 expression. S100A7 mediates events that regulate EGF-induced migration. Loss of S100A7 expression alters the phosphorylation state of receptors and inhibits growth factor-induced cell motility and invasion on fibronectin-coated plates and matrigel-coated chambers. In addition, downregulation of S100A7 decreases angiogenesis in matrigel assays. There were definitive osteolytic lesions observed in mice injected with control cells as compared to those injected with the S100A7-downregulated cells, and our *in vitro* data on the increased size and number of TRAP+ osteoclasts supports the above results. Furthermore, cytokine analysis revealed increased expression of IL-8, an osteolytic factor, in the supernatants of control cells as compared to S100A7-downregulated cells. This study suggests that S100A7 plays an important role in EGFR-mediated signaling and osteoclast formation in breast cancer.

## Materials and Methods

### Cell culture and reagents

MCF-7 and MDA-MB-468 cells were obtained from American Type Culture Collection (ATCC). The MCF-7 and MDA-MB-468 cells were grown in DMEM with 10% fetal bovine serum and with 1% penicillin/streptomycin. Stable S100A7-downregulated MDA-MB-468 cells and vector control cells were obtained from Dr. Kornelia Polyak from Dana-Farber Cancer Institute (Boston, MA) and were maintained in McCoy's medium with 10% FBS, 1% penicillin/streptomycin, and 2 µg/ml of puromycin. MCF-7 cells were maintained in DMEM media with 10% FBS, and 1% penicillin/streptomycin. The cells were grown in a humidified atmosphere of 5% CO_2_ at 37°C. Cells were seeded in 75 cm^2^ flasks with 15 ml of growth medium, unless otherwise mentioned. All our experiments were done using MDA-MB-468 cell lines except for Figure 1A, where MCF-7 cell lines were used. Antibodies were obtained as follows: anti-p-Tyr-EGFR1173/p-Tyr1248, p-Tyr-Src 416, p-Tyr-SHP2, and β-Actin were all obtained from Cell Signaling Technology Inc (Danvers, MA) and anti-p-Tyr-EGFR1173 as well as anti-EGFR were from Santa Cruz Biotechnology (Santa Cruz, CA). Antibody to S100A7 was from Imgenex Corp (San Diego, CA). EGF was obtained from Peprotech Ltd (Rocky Hill, NJ).

### Stimulation of cells

MCF-7 and MDA-MB-468 cell lines were grown in 6-well plates until 80% confluent. The cells were then washed twice with phosphate-buffered saline (PBS) and were subsequently starved in 0.5% serum overnight at 37°C. The serum-starved cells were stimulated with various concentrations of EGF at 37°C for 30 minutes. At the end of the stimulation, cells were washed with PBS and lysed in modified radioimmune precipitation assay buffer (RIPA) (50 mM Tris-HCl, pH 7.4, 1% Nonidet P-40, 150 mM NaCl, 1 mM phenylmethylsulfonyl fluoride, 10 µg/ml aprotinin, 10 µg/ml leupeptin, 10 µg/ml antipain, 10 µg/ml chymostatin, 100 mg/ml TI, 10 µg/ml pepstatin, 10 µM sodium vanadate, 10 mM sodium fluoride and 10 mM sodium pyrophosphate). The lysate was used immediately or stored at −80°C until further use.

### Immunoblotting

For the immunoblotting, aliquots of cell extracts containing equal amounts of protein were resolved by 4–12% sodium dodecyl sulphate electrophoresis (SDS-PAGE) (Invitrogen, Carlsbad, CA) and electroblotted onto nitrocellulose membranes (Bio-Rad, Hercules, CA).

### Cell migration assays

Briefly, MDA-MB-468 cells were washed twice in PBS with 0.5% serum and then resuspended in 0.5% serum containing McCoy's medium. A 24-well plate containing 8 µm porosity inserts (Costar Corporation, Kennebunk, ME) was used for this experiment to detect MDA-MB-468 cell chemotaxis. The inserts were precoated with fibronectin (25 µg/ml), and McCoy's Medium (600 µl) containing EGF (20 ng/ml) was added to the bottom well. A volume of 150 µl (containing 1×10^5^ MDA-MB-468 cells) from each sample was loaded onto the upper well. The plates were next incubated for 8 hours at 37°C in 5% CO_2_. After incubation, the porous inserts were removed carefully, then cells on the inner surface were wiped with a cotton swab and cells on the lower surface of the membrane were stained with the Diff-Quik Stain kit (Dade Diagnostics, Puerto Rico). The surface was allowed to dry and the number of migrated cells was counted in at least seven different fields. The results are expressed as the percent of migrated cells as compared to the control (untreated cells). Each experiment was performed three times in triplicate.

### Chemoinvasion assay

Briefly, MCF-7 cells were detached from the plates with 10 mM EDTA in PBS, washed twice, and then suspended at 2.5×10^6^ cells/ml in McCoy's medium containing 0.5% FBS. A 24-well plate containing a matrigel coating (Becton Dickinson Biosciences) was used for this experiment. Cells (150 µl) from each sample were loaded onto the upper well. The lower well contained 600 µl of 0.5% FBS McCoy's medium containing growth factor (EGF, 20 ng/ml). The plates were incubated for 16 hours at 37°C in 5% CO_2_. After incubation, the inserts were removed carefully and the cells were fixed and stained using the Diff-Quik Stain kit (Dade Diagnostics). The results are expressed as the percent of migrated cells as compared to the control (untreated cells).

### Culture for osteoclast formation and Transwell assays

Bone marrow cells were obtained from 6 week-old mice after flushing the long bones and passage of the cells through a 40 µm nylon mesh. The cells were then washed and incubated in tissue culture dishes at 37°C in 5% CO_2_ in the presence of M-CSF (10 ng/ml). After 24 hours in culture, the nonadherent cells were collected and layered onto Histopaque 1088 (Sigma Aldrich Inc.). Cells at the gradient interface were collected, washed, and plated in 24-well tissue culture plates at 5.0×10^5^ cells/well. After 6 hours, transwell inserts (8 µm pore size, Corning) containing S100A7 vector control cells or S100A7-downregulated cells (5×10^4^ cells/well) were placed in wells containing precursor osteoclasts. Treatment with RANKL (20 ng/ml) without breast cancer cells served as a positive control. TRAP staining was performed with the Leukocyte Phosphatase Kit (Sigma Aldrich Inc.).

### Animal model of metastasis

Female CB-17 SCID mice (4 weeks-old) were obtained from Charles River Laboratories (Boston, MA) and maintained in a specific pathogen-free barrier animal facility for 1 week prior to the start of the experiments. MDA-MB-468 cells (S100A7-expressing control cells and S100A7-downregulated cells) were grown to 70% confluence, and the culture medium was changed to that without antibiotics 24 hours prior to harvesting. Single suspensions of cells expressing S100A7 (vector control) or S100A7-downregulated cells were harvested from the subconfluent cultures by exposure to 0.25% trypsin and 0.02% EDTA. Trypsinization was stopped by the addition of medium containing 10% FBS (without antibiotics). Cells were kept at 4°C until use (0–1 hour). Cell concentration and viability (Trypan blue exclusion, >90% viability) were determined by a hemocytometer. SCID mice were anesthetized using Avertin (2.5%), 0.02 mg/kg of bodyweight. The animal was then shaven over the lower end of the femur and upper half of the tibia and the injection site prepped with betadine scrub followed by the use of a 70% alcohol wipe. A 1-cm skin incision was made on the antero-medial part of the leg and the muscle was moved using blunt forceps. The bone was drilled using a microdrill (Roboz Surgical Instruments) with a 0.06 mm caliber point (vWR Laboratories). Single suspensions of cells expressing S100A7 or S100A7-downregulated cells (5×10^4^) in a final volume of 10 µl were injected into the upper 1/3 of the tibia at a tilted angle of 45° in the two groups of mice. To prevent leakage of the cells into surrounding muscles, the injection site was sealed with surgical bone wax (vWR Laboratories). The wax was allowed to solidify and the skin was sutured back using black silk. The animals tolerated the procedure well and no anesthesia-related deaths occurred during the procedure. One mouse, which acquired a postoperative infection at the suture site, was treated with betadine washes and daily dressings with triple antibiotic ointment, and recovered well. Buprenorphine was used as an analgesic (0.05 mg/kg/body weight subcutaneously) twice daily for 3–4 days. Mice from both groups were anesthetized by inhalation using 1.5% isofluorane in 98.5% oxygen, and Micro-CT scanning was done using a GE eXplore Micro-CT scanner (GE Healthcare Ltd, UK) at 93 µm resolution 6 weeks after injection. The mice were then euthanized using CO_2_ inhalation, the tibias were dissected out and scout view radiographs taken with a micro-CT40 scanner at 12 µm resolution from SCANCO Medical AG (Switzerland).

### Tumor harvesting and preparation

The mice were euthanized by CO_2_ inhalation on the 8^th^ week and the tibias were resected from both groups of mice, and then placed in 4% paraformaldehyde at 4°C for 2 days. Micro-CT scanning with high resolution was done on the bones to confirm the results of the *in vivo* imaging, which was done at low resolution. After the scanning, tumors from both groups of mice were transferred to decalcifying solution for 7 days at room temperature. Preparation and staining of frozen sections of the tibias were done at our Institute's microscopic histology core facility. The paraffin sections were analyzed by H & E staining.

### Angiogenesis assay

4 week-old CB-17 SCID mice were purchased from Charles River Laboratories. Growth factor-reduced matrigel was obtained from BD Biosciences (Bedford, MA), and thawed overnight at 4°C. The required amount was appropriately calculated and the remaining aliquots were stored at −80°C. The gel was mixed at a ratio of 5×10^6^ cells for 500 µl of matrigel. The mixture was implanted subcutaneously into two sites at the midline and the needle was retained for 1–2 minutes to allow the plug to solidify. The needle was subsequently removed by slow rotation to ensure that the matrigel did not flow out. After 3 weeks, the mice were anesthetized using Avertin (2.5%), 0.02 mg/kg of bodyweight and the matrigel plug was removed. Mice were euthanized with CO_2_, and then 0.5 ml to 2 ml of blood was collected from the heart and placed into heparin-coated tubes for estimation of systemic hemoglobin. One plug was removed, dipped in embedding medium (Tissue Tek) and snap-frozen on dry ice. Immunohistochemistry for CD31 was done on the embedded specimens at the microscopy histology core facility at our Institute. The other plug was weighed and dissolved in physiological saline (0.9% NaCl). The tissue was grounded using a Wheaton tissue grinder and the supernatant was analyzed for hemoglobin content using a Hemoglobin Assay Kit (containing Drabkin's reagent) from Sigma Diagnostics. The results were interpreted as blood volume per unit weight of matrigel.

### Cytokine analysis

The conditioned medium was centrifuged at 14,000 rpm/min at 4°C to allow the cells and debris to settle down. The upper ¾^ths^ of the sample was removed and analyzed for human IL-8 using an ELISA kit (R & D Systems).

### Statistical analysis

The results are expressed as the means +/− S.D. of data obtained from three experiments performed in triplicate. Statistical significance was determined using the Student's *t*-test.
